# Supplementary material for: Disability-adjusted life years (DALYs) based COVID-19 health impact assessment: A systematic review protocol
Source: PLoS One. 2022 Sep 12;17(9):e0274468. doi: 10.1371/journal.pone.0274468 (PMC9467350; doi:10.1371/journal.pone.0274468)
Supplement: S1 Text — (PDF) [file pone.0274468.s002.pdf]

Searching strategy for each database.

1. Scopus

(**All fields** (Impact) OR **Article title, abstract, keywords** (Burden) AND **All fields** ("COVID-19") OR **Article title, abstract, keywords** ("COVID 19") OR **Article title, abstract, keywords** ("SARS-COV-2") OR **Article title, abstract, keywords** ("SARS COV 2") OR **Article title**, **abstract, keywords** ("Coronavirus disease 2019") OR **Article title, abstract, keywords** ("Coronavirus disease-19") OR **Article title, abstract, keywords** ("Coronavirus diseases 19") OR **Article title, abstract, keywords** ("Severe acute respiratory syndrome coronavirus 2") OR **Article title, abstract, keywords** ("Severe acute respiratory syndrome coronavirus-2") OR **Article title, abstract, keywords** ("Novel coronavirus") OR **Article title, abstract, keywords** ("Wuhan coronavirus") AND **All fields** (Health) AND **All fields** (DALY) OR **Article title, abstract, keywords** (DALYs) OR **Article title, abstract, keywords** ("Disability-adjusted life years"))

2. PubMed

(**All fields** (Impact) OR **Title/Abstract** (Burden) AND **All fields** ("COVID-19") OR **Title/Abstract** ("COVID 19") OR **Title/Abstract** ("SARS-COV-2") OR **Title/Abstract** ("SARS COV 2") OR **Title/Abstract** ("Coronavirus disease 2019") OR **Title/Abstract** ("Coronavirus disease-19") OR **Title/Abstract** ("Coronavirus diseases 19") OR **Title/Abstract** ("Severe acute respiratory syndrome coronavirus 2") OR **Title/Abstract** ("Severe acute respiratory syndrome coronavirus-2") OR **Title/Abstract** ("Novel coronavirus") OR **Title/Abstract** ("Wuhan coronavirus") AND **All fields** (Health) AND **All fields** (DALY) OR **Title/Abstract** (DALYs) OR **Title/Abstract** ("Disability-adjusted life years"))

3. Web of Science

(**All fields** (Impact) OR **Title** (Burden) AND **All fields** ("COVID-19") OR **Title** ("COVID 19") OR **Title** ("SARS-COV-2") OR **Title** ("SARS COV 2") OR **Title** ("Coronavirus disease 2019") OR **Title** ("Coronavirus disease-19") OR **Title** ("Coronavirus diseases 19") OR **Title** ("Severe acute respiratory syndrome coronavirus 2") OR **Title** ("Severe acute respiratory syndrome coronavirus-2") OR **Title** ("Novel coronavirus") OR **Title** ("Wuhan coronavirus") AND **All fields** (Health) AND **All fields** (DALY) OR **Title** (DALYs) OR **Title** ("Disability-adjusted life years"))
