# Supplementary material for: Disability-adjusted life years (DALYs) based COVID-19 health impact assessment: A systematic review protocol
Source: PLoS One. 2022 Sep 12;17(9):e0274468. doi: 10.1371/journal.pone.0274468 (PMC9467350; doi:10.1371/journal.pone.0274468)
Supplement: Attachment — Submitted filename: PONE-D-22-15834_Revision.pdf [file pone.0274468.s003.pdf]

The protocol covers an interesting and really actual topic regarding the review of DALYs-based health impact publications. Although the manuscript is almost technically sounding, there is still room to be improved. First of all, the rationale section (lines 65-69) must be deepened adding more literature about the health impact assessment using DALYs. Thus, it should be useful to add some scientific evidence for each geographical macro-area to provide an overall state of the art and to better support the rationale of the study. Few examples are:

*"Gianino MM et al. Burden of COVID-19: disability-adjusted life years (DALYs) across 16 European countries. European Review for Medical and Pharmacological Sciences. 2021 Sep 1;25(17):5529-41."*

*"Zhao J, et al. Disease burden attributable to the first wave of COVID-19 in China and the effect of timing on the cost-effectiveness of movement restriction policies. Value Health. 2021; in press. doi: 10.1016/j.jval.2020.12.009."*

*"Pifarré i Arolas H, et al. Years of life lost to COVID-19 in 81 countries. Sci Rep. 2021;11:3504. doi: 10.1038/s41598-021-83040-3"*

Moreover, authors should rephrase the inclusion and exclusion criteria in the eligibility criteria subsection. In particular, the exclusion criteria are not the exact contrary of the inclusion ones. Authors could also expand their search strategy to additional scientific databases as CINAHL and EMBASE.
In addition to the search terms, it is useful to add, as a supplementary files, the whole search string, one for each database.
The present reviewer does not know if the authors already thought about the provision of a quantitative synthesis of the results (i.e., meta-analysis) in the final systematic review. It would be interesting to read the results of a meta-analysis of the publications reporting health impact assessment based on DALYs. From a technical point of view, it seems feasible given the presence of a common indicator (i.e., DALY) across the studies that will be included and the statistical properties that a continuous variable has.
Lines 174-176 should be moved in the discussion section.
Authors should consider expanding the discussion of the protocol's limitations.
